# From Extractive to Inclusive Health Preservation Model

**DOI:** 10.17691/stm2025.17.2.01

**Published:** 2025-04-30

**Authors:** N.N. Karyakin

**Affiliations:** Associate Professor, Rector; Privolzhsky Research Medical University, 10/1 Minin and Pozharsky Square, Nizhny Novgorod, 603005, Russia

**Keywords:** health preservation, extractive model, inclusive model, individual health development program, healthcare system, functional dispensarization

## Abstract

Nowadays, the healthcare systems of several countries have reached an efficiency threshold, therefore, even increased fundin will not significantly impact life expectancy, particularly in terms of healthy life expectancy. This situation underscores the necessity of complementing national healthcare systems, which focus on early diagnosis and treatment of diseases, with a multi-level primary prevention system that extends beyond the traditional boundaries of healthcare.

In this regard, the inclusive model has been proposed, focused on health preservation, in contrast to the extractive model that emphasizes the exploitation of biological organism reserves. This inclusive model is represented as an “onion bulb” structure, where each layer consists of a set of interconnected measures within a multi-level policy aimed at strengthening and maintaining the population health.

## Introduction

In modern developed countries, the population growth and rising urbanization have led to an increase in healthcare expenditures, driven both by a growing number of elderly citizens and the rise and “rejuvenation” (epidemiological transition) of chronic non-communicable diseases. This creates an additional financial burden on families and the state, reducing the quality of life. In fact, the healthcare costs of key developed countries have increased on average tenfold during the XX century, rising from 0.5–1 to 10–11% of gross domestic product [1].

Over the past few centuries, the Earth’s population has changed dramatically. Historical demographers estimate that around 1800, there were only about 1 billion people [2]. The world population growth rate peaked in 1962–1963 with 2.2% per year [3]. Since then, the growth rate has halved, yet the total number of planet inhabitants has reached 8 billion [4].

The global average life expectancy has doubled over the last 150 years. Until the XX century, it did not exceed 35–45 years [5, 6]. Alongside the increase in life expectancy, the costs associated with maintaining it have also risen. The cost of each additional year of life has increased tenfold over the last 50 years — from 100– 200 to 1500 euros — and continues to rise. Particularly expensive is the provision of healthcare for patients in the older age group: while average healthcare costs for individuals under 50 years old are less than 1000 euros per year, for patients aged 65 it is already 2500 euros, for those aged 70 it can reach up to 4500 euros, and for individuals aged 80, it can be 14,000 euros [7].

Researchers believe that the current healthcare system in several countries has reached its maximum effectiveness, and even if more money is spent on it, there will be no significant additional impact on life expectancy [8–14].

All of the aforementioned necessitate a reassessment of the prior emphasis in medicine, which primarily targeted disease. The focus should shift from overall life expectancy to healthy life expectancy and the prevention of mortality in the working-age population.

Therefore, **the aim of this study** is to propose an inclusive health preservation model to be implemented at the national level, designed to stimulate and support the biological organism reserves through interconnected multi-level policy measures.

## Transition from the extractive to the inclusive health preservation model

The current healthcare model, which we have termed *extractive*, operates based on the exploitation of the biological organism reserves. In contrast, it is advisable to propose an *inclusive* model built on the development of existing biological reserves, as well as their prosthetics using new biotechnological solutions.

Each of the models under consideration, in our opinion, includes the following four groups of factors.

Individual factors represent a set of factors related to mental and physical health that the individual is able to manage independently to a large extent; they form the model of individual health-saving behavior.

Social (collective) factors are implemented at the level of legal entities and social groups of which the individual is a part or enterprises that produce goods and services directly affecting human health (fitness and wellness industries, producers and retailers of food products, cosmetics, and other goods and services).

Governmental factors encompass a system of measures implemented through instruments of authority, including regulatory, financial, institutional, etc.

Environmental factors are classified as anthropogenic and non-anthropogenic; the latter go beyond the scope of this work.

This approach differs from the previously accepted in the scientific literature, which distinguishes between public and individual health, often limiting the “public” level exclusively to the description of healthcare systems [15–18].

It is possible that in the future, a separate level of health study will be established as the “family level”, as it is difficult to envision one family member being healthy while other household members have a low health level. In our opinion, this idea requires separate consideration and development.

The modern individual often adopts a passive (infantile) attitude towards their health. This is largely determined by the broadly implemented social policies of the state, which carries the financial burden of stopping destructive behaviors exhibited by individuals, along with the low level of personal responsibility regarding one’s health. For example, smoking, alcohol consumption, or drug use are considered personal matters, while the treatment of conditions provoked by these factors is seen as the responsibility of the state?! Another example is the following: a patient is diagnosed with familial dyslipidemia (a genetically determined condition characterized by elevated blood cholesterol levels); the patient undergoes examination and is informed about their condition, receiving recommendations; however, they do not change their dietary and physical activity patterns and are unable to fundamentally transform their lifestyle, which exacerbates the disease; ultimately, this leads to early myocardial infarctions or other diseases, the treatment of which is fully covered by public healthcare.

Analysis of trends in the medicalization of society [19–23] indicates that the regular use of pharmaceuticals begins at an increasingly younger age and is often perceived as the “only” way to recovery and health improvement. Pharmacotherapy is frequently regarded by individuals as a simple and convenient solution to health issues, in contrast to the multifaceted healthpreserving behavior.

Yet, currently, in many countries around the world, including Russia, a segment of the population is emerging that values their health and, with varying degrees of success, engages in health-preserving behaviors [24–31]. This group primarily consists of individuals over the age of 40, who possess a certain level of wealth and higher education. However, this demographic is not large — less than 30% of the target group. To clarify, we believe that approximately 40 years of age is a key age in the inclusive health preservation model! Throughout history, humans lived active lives typically until the age of 35–40, consequently, our bodies have adapted to cope with aggressive factors during this period. Beyond this age, sanogenic/compensatory mechanisms slow down, and the body’s tolerance to various biological and social stresses diminishes. If an individual does not consciously begin to take care of their health, subclinical manifestations accumulate rapidly, followed by the onset of the first symptoms of diseases in various target organs, which differ for each individual.

Individual sanogenic factors (such as physical activity, nutrition, sleep, and many others) have been extensively studied both empirically and scientifically [32–37]. All of these factors contribute to conscious health-preserving behaviors, which largely determine the duration of healthy life.

However, why is this well-known set of actions not widely implemented? In our opinion, there are several reasons.

Firstly, the extractive model lacks a methodology for a comprehensive assessment/diagnosis of an individual’s health level, which we propose to call “*functional dispensarization”*. (Everything that is not measurable does not exist!) Based on its results, it will be possible to formulate an *individual health development program* (IHDP), therefore, consciously changing behavior, including consumer priorities. Adopting this approach will lay the groundwork for establishing public institutions that implement these tools and will stimulate the production of necessary goods and services that promote health development^1^. However, it is evident that this approach will not be relevant for everyone. In our opinion, it is possible to outline a path to health and create the conditions to follow it, but each individual must walk that path independently.

It is important not to confuse functional dispensarization with medical care aimed at the early diagnosis of diseases. In the first case, we study standardized load tolerance and the organism’s physiological capabilities to adapt; in the second case, we look for early signs of morphological changes indicating reversible or irreversible diseases.

Even after severe illnesses, such as cerebrovascular disorder, acute coronary syndrome, or coxarthrosis leading to endoprosthesis, etc. (after the acute phase), there remains the possibility for comprehensive organism development. Moreover, a person who has experienced a serious illness already possesses high motivation, namely, the extension of their own life, which was threatened. However, there are currently very few specialists capable of comprehensively assessing an individual’s health considering past/accumulated diseases and prescribing IHDP, and in some places, they are entirely absent.

Secondly, in the extractive model of health management, the medical and pharmaceutical industries, as well as pharmacy networks, benefit from the increasing number of patients requiring high-cost treatment methods, thereby ensuring a guaranteed sales market.

The work of medical organizations is also associated with the inflow of funds for the treatment of complex and severe patients. Consequently, these stakeholders of the health-preserving system are unlikely to initiate processes leading to a transition toward preventive medicine. However, unless the issue is fundamentally restructured, the hospital level of medical care will eventually become overloaded, and healthcare staff will continue to migrate from outpatient settings to inpatient facilities, further exacerbating the situation.

Based on the aforementioned, citizens who would like to maintain their health rather than treat illnesses are deprived of the opportunity to receive professional consultation in the field of health preservation and enhancement.

The solution to this problem lies in integrating preventive medicine physicians into the healthcare system, whose activities should not be limited to promoting healthy lifestyles and identifying early signs of disease. In some countries, such a position does not exist at all. The role of this specialist is to conduct a comprehensive examination of the individual, determine their readiness for active management of their own health, and develop an IHDP. It is evident that this approach will require a reformation of the healthcare organization system, the establishment of an economically justified tariff within the medical insurance framework, the creation of conditions to attract private investments, and the development of infrastructure that is accessible to citizens’ residences. Additionally, there is a need to ensure the market is supplied with necessary goods and services while simultaneously restricting the circulation of products and services that have proven negative effects on human health. Furthermore, there has to be a training of a broad base of qualified specialists, primarily those with medical education. Thus, within the inclusive health preservation system, every healthcare worker should possess competencies in health management (including physicians and nurses of various specialties). However, the core of the system will consist of specialists directly engaged in health-related issues, namely preventive medicine physicians, working alongside their assistants, who may include non-medical personnel such as health management instructors/specialists who have undergone appropriate training.

The foundation of this approach should be a vertically integrated training program for physicians and nurses with competencies in health preservation. In this regard, the active participation of students in diagnosing their own health, developing an IHDP, and implementing it with subsequent evaluation of the achieved results plays a crucial role. There is a need to train educators for medical colleges and universities who apply this approach through personal experience and possess the necessary educational technologies.

However, are universities and colleges currently training physicians or nurses to be capable of assessing health status and developing an IHDP? From the perspective of a medical university employee, I can say that no. This is understandable. Society and medical technology manufacturers have not previously prioritized the training of physicians capable of health management. Our healthcare system is rooted in the XX century, when the primary focus was on treating the sick.

**Figure 1.**
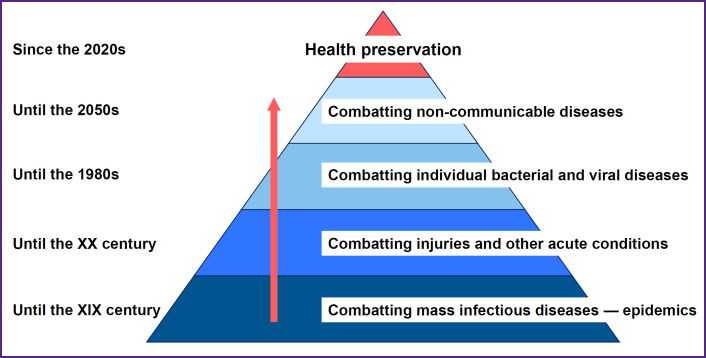
The healthcare priorities evolution stages

The healthcare priorities evolution stages shown in Figure 1 represent a natural progression of European thinking regarding health preservation. In the first stage, combating mass infectious diseases is the main priority. The second stage emphasizes treating injuries and other acute conditions. The third stage concentrates on curing individual bacterial and viral diseases. The fourth stage focuses on addressing non-communicable diseases. Only by achieving these objectives and accumulating sufficient knowledge about health and pathology, including intermediate/borderline conditions between health and disease and vice versa, can a qualitative transition to a new paradigm of healthcare occur, which is part of the inclusive health preservation model. Therefore, this issue is being systematically raised at this time. It is noteworthy that the healthcare model based on primary prevention and health maintenance, developed by an academician N.A. Semashko, has always been a priority for Soviet and Russian medicine. However, the country’s transition from a socialist model to a capitalist one has led to the widespread implementation of profitgenerating mechanisms, starting from manufacturers of pharmaceuticals and medical devices to providers of medical services (such as hospitals). What is the easiest way for a legal entity to generate revenue: through the treatment of disease or through its prevention and the patient health maintenance? Who will pay for health? What criteria should be used to assess outcomes? Many questions remain regarding the transition to a new healthcare model. Finding answers is crucial for researchers engaged in designing the inclusive health preservation model.

In addition to the medical component, health is influenced by a wide range of various factors. These include:

the low availability of food products enriched with probiotics, dietary fibers, vitamins, and other beneficial components, alongside the presence of food products and other goods with potentially harmful effects on human health (such as tattooing substances, smoking mixtures, and other materials that come into contact with the human body), which is particularly relevant for children;
the low consumers’ commitment to choosing “functional/green” products and other health-preserving behaviors, due to both a lack of relevant knowledge and a disregard for personal health responsibility;
the evident anthropogenic disruption of ecological balance in the XX and XXI centuries received little attention from society just 100 years ago. In many countries today, economic criteria often take precedence over health and the ecological legacy that we, as a generation, will pass on to our descendants.

All of these factors are closely related to the health of each individual and should find their place in the inclusive health preservation model creation.

Certainly, the government is implementing various measures to strengthen the population health, with each agency and authority level executing corresponding health programs. However, this occurs in the absence of interagency coordination and collaboration. The activities of governmental institutions, primarily at the regional level (such as the Ministry of Health, Ministry of Sports, Ministry of Education, and Ministry of Social Protection), as well as of public and private organizations regarding health preservation, are not synchronized with one another. This lack of coordination hinders the establishment of a multi-component health-preserving environment around individuals, resulting in inefficiencies at the population level due to the fragmented implementation of solutions. For instance, there is a lack of close collaboration between the production and sale of “functional/green” food products and healthcare; between social support for the elderly and the fitness industry; and between the work of sports centers under the Ministry/Department of Sports and healthcare. The range of measures aimed at promoting goods and services for health development remains limited.

In many countries, a governmental system for mental health support has not been developed: there is a lack of regulatory frameworks concerning psychological well-being; there are no governmental tools or solutions aimed at providing psychological assistance to children and adults; there are also insufficient financial resources allocated for these purposes. The psychological support provision is primarily limited to medical care in situations requiring psychiatric intervention. In recent years, Russia has made efforts to increase the number of psychologists in medical and educational institutions; however, this requires a comprehensive approach and additional funding. Meanwhile, societal demand for these services is growing.

**Figure 2.**
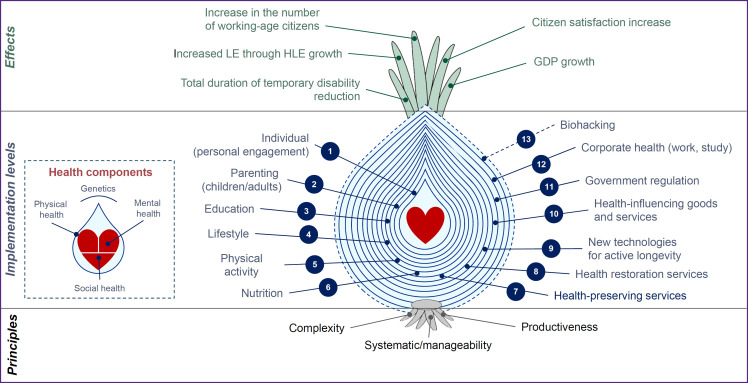
“Onion bulb” structure of the inclusive health preservation model LE — life expectancy; HLE — healthy life expectancy; GDP — gross domestic product

Thus, the creation of the inclusive health preservation model is only possible through the implementation of a synchronized multi-level policy based on complexity and productivity. We have conceptualized this model as an “onion bulb”, with each layer interconnected with others and representing a set of measures aimed at addressing the issues related to this layer (Figure 2).

At this stage of studying health preservation, we have identified 12 layers, plus one additional layer being biohacking, which encompasses a set of medical and biotechnological solutions that, based on specific effect on the human body, increase a healthy life expectancy.

The design of each layer, the study of their interconnections, and many other organizational issues related to health preservation at national levels will continue to be subjects of discussion among scholars and healthcare organizers for an extended period. The important thing is that the question has already been raised.

## Conclusion

With consideration to modern realities, a priority task is to increase the healthy life expectancy. In this regard, the inclusive model has been proposed, focused on health preservation, in contrast to the extractive model that emphasizes the exploitation of biological organism reserves. This inclusive model is represented as an “onion bulb” structure, where each layer consists of a set of interconnected measures within a multi-level policy aimed at strengthening and maintaining the population health.

## Footnotes

^1^We deliberately use the term “health development” because health is a dynamic process, and each individual's current health level is not their maximum; everyone has the potential to improve it.
